# Adherence to Highly Active Antiretroviral Treatment in HIV-Infected Rwandan Women

**DOI:** 10.1371/journal.pone.0027832

**Published:** 2011-11-17

**Authors:** Stephenson Musiime, Fred Muhairwe, Alfred Rutagengwa, Eugene Mutimura, Kathryn Anastos, Donald R. Hoover, Shi Qiuhu, Elizaphane Munyazesa, Ivan Emile, Annette Uwineza, Ethan Cowan

**Affiliations:** 1 King Faisal Hospital, Kigali, Rwanda; 2 Byumba District Hospital, Gicumbi District, Northern Province, Rwanda; 3 Faculty of Medicine, National University of Rwanda, Huye, Rwanda; 4 Women's Equity in Access to Care and Treatment (WE-ACTx), Kigali, Rwanda; 5 Albert Einstein College of Medicine and Montefiore Medical Center, Bronx, New York, United States of America; 6 Rutgers, The State University of New Jersey, New Brunswick, New Jersey, United States of America; 7 School of Health Sciences and Practice, New York Medical College, Valhalla, New York, United States of America; 8 National Reference Laboratory, Kigali, Rwanda; 9 Rwanda Biomedical Center, Kigali Health Institute, Kigali, Rwanda; 10 Faculty of Medicine, National University of Rwanda, Huye, Rwanda; 11 Albert Einstein College of Medicine, Department of Emergency Medicine, Department of Epidemiology & Population Health, Bronx, New York, United States of America; University of Toronto, Canada

## Abstract

**Background:**

Scale-up of highly active antiretroviral treatment therapy (HAART) programs in Rwanda has been highly successful but data on adherence is limited. We examined HAART adherence in a large cohort of HIV+ Rwandan women.

**Methods:**

The Rwanda Women's Interassociation Study Assessment (RWISA) was a prospective cohort study that assessed effectiveness and toxicity of ART. We analyzed patient data 12±3 months after HAART initiation to determine adherence rates in HIV+ women who had initiated HAART.

**Results:**

Of the 710 HIV+ women at baseline, 490 (87.2%) initiated HAART. Of these, 6 (1.2%) died within 12 months, 15 others (3.0%) discontinued the study and 80 others (19.0%) remained in RWISA but did not have a post-HAART initiation visit that fell within the 12±3 month time points leaving 389 subjects for analysis. Of these 389, 15 women stopped their medications without being advised to do so by their doctors. Of the remaining 374 persons who reported current HAART use 354 completed the adherence assessment. All women, 354/354, reported 100% adherence to HAART at the post-HAART visit. The high self-reported level of adherence is supported by changes in laboratory measures that are influenced by HAART. The median (interquartile range) CD4 cell count measured within 6 months prior to HAART initiation was 185 (128, 253) compared to 264 (182, 380) cells/mm^3^ at the post-HAART visit. Similarly, the median (interquartile range) MCV within 6 months prior to HAART initiation was 88 (83, 93) fL compared to 104 (98, 110) fL at the 12±3 month visit.

**Conclusion:**

Self-reported adherence to antiretroviral treatment 12±3 months after initiating therapy was 100% in this cohort of HIV-infected Rwandan women. Future studies should explore country-specific factors that may be contributing to high levels of adherence to HAART in this population.

## Introduction

The Human Immunodeficiency Virus (HIV) epidemic has had a devastating impact on sub-Saharan Africa which contains four countries with the world's highest HIV prevalence.[1] As scale-up highly active antiretroviral treatment (HAART) treatment programs increase and HAART becomes more available in sub-Saharan countries, the quality of life for people living with HIV infection is progressively improving. However, optimal adherence to HAART is essential for better well-being and increased life expectancy of HIV-infected individuals accessing treatment and reduced transmission of HIV. The need for high adherence to treatment is clearly important in countries such as Zimbabwe, Botswana and Swaziland where HIV infected individuals make up as much as 23-26% of the population.[1] But even in other sub-Saharan countries such as the Comoro islands, Madagascar, Mauritius and Rwanda where HIV prevalence rates are lower and scale-up of antiretroviral treatment programs has been successful, adherence is an essential factor for optimal HIV health outcomes.

Poor adherence to HAART can lead to HIV disease progression, evolution of drug resistance, and subsequent immunological and clinical failure.[2], [3] Adherence to antiretroviral therapy is essential for maximal suppression of viral replication and is believed to be a critical determinant of long-term survival among HIV-infected individuals.[4], [5] Non-adherence to these medications can greatly lower their effectiveness resulting in poor therapeutic outcomes.[2], [3] More than 95% of the prescribed antiretroviral treatment doses should be taken for optimal response; lesser adherence is often associated with virologic failure.[6] Recent studies demonstrate that HIV-infected individuals who adhere to HAART have HIV viral load reduced to levels so low that HIV transmission is dramatically reduced or eliminated.[7] Thus, the benefits of adherence to HAART make it critical to identify factors associated with non adherence that could lead to increased HIV incidence, treatment failure and drug resistance.

One meta-analysis of adherence to HAART in sub-Saharan Africa and North America estimated that only 55% of persons in the populations studied achieved adequate levels of adherence.[8] Unfortunately, the studies of adherence in sub-Saharan African populations used in this meta-analysis were primarily conducted in patients with early access to limited therapy and further suffered from small sample sizes. One study in five East African countries found that less than 50% of facilities routinely measured patient adherence to HAART and only 25% of these facilities calculated adherence rates for the clinic population as a whole.[9] In a high risk urban population in sub-Saharan Africa 38% of patients were non-adherent to HAART at some point during their treatment.[10] Furthermore, adherence to HAART appears to decrease steadily over time but may vary by gender and HAART regimen.[11] In Rwanda, where HIV prevalence is 2.9% and approximately 70,047 HIV+ persons receive HAART, there has been limited study of both HAART adherence rates and factors associated non-adherence. [12] This study seeks to improve knowledge of HAART adherence in sub-Saharan Africa by examining adherence to HAART in a large cohort of women enrolled in the Rwanda Women's Interassociation Study and Assessment (RWISA).

## Methods

### Design

We analyzed data collected from participants in RWISA, an observational prospective cohort study that assessed effectiveness and toxicity of HAART. RWISA enrolled participants in 2005 with follow-up visits every six months through 2008. Methods in the RWISA study have been previously described in detail elsewhere.[13] At baseline, there were 710 HIV-positive women.

#### Subjects

Women were recruited from the Women's Equity in Access to Care and Treatment (WE-ACTx) clinical site in Kigali, community- based organizations, and other HIV clinical care sites in Kigali. To be eligible participants had to be 25 years of age or older, willing to undergo voluntary counseling and testing for HIV, able to complete an interview in the local Kinyarwanda language and lived in Rwanda in 1994. All HIV+ women were antiretroviral naïve at enrollment. Exposure to single-dose nevirapine to prevent mother to child transmission of HIV was not an exclusion criterion. Women were scheduled to initiate HAART once clinical or laboratory indications identified that they were eligible by Rwandan national standards. The study protocol and its consent process were approved by the Rwanda National Ethics Committee and the Institutional Review Board of Montefiore Medical Center, Bronx, NY USA. This analysis was restricted to women who initiated HAART by their fifth RWISA visit (within ∼2 years) after enrolling in RWISA and returned for a follow up visit 12±3 months after HAART was initiated.

#### HAART distribution

All HAART medications were distributed by the central pharmacy housed at the site of the participant's care. First line HAART medications included stavudine (d4T), lamivudine (3TC), and nevirapine (NVP), zidovudine (AZT), and efavirenz (EFV). The most common second line medications were abacavir (ABC) and lopinavir/ritonavir.[13] The pharmacy distributed a one-month supply of medications to patients receiving HAART. Patients returned to the clinic monthly to refill their medications. Patients who failed to return to the clinic had a follow-up home visit scheduled, as required by the Rwandan national guidelines.

#### Clinical Laboratory Measurements

CD4 counts were measured at the National Reference Laboratory of Rwanda by FACS Count (Becton Dickinson, San José, CA). Other laboratory measures were performed at King Faisal Hospital using standard methods.

#### Data collection procedures

At baseline and each subsequent visit, participants underwent a physical examination. Medical and social history was also collected, including demographic characteristics and questions regarding the exact date of HAART initiation (verified by clinic data) and adherence to HAART as described below. Specimens were taken for measuring CD4 cell count, full blood count and other laboratory studies. A trained interviewer re-assured each participant about strict confidentiality regarding response to HAART adherence questions and that their responses would not negatively impact their clinical care. For the analyses presented here, information on adherence was obtained from all RWISA interviews conducted 12±3 months after the participant had initiated HAART. If multiple visits from the same person fell into this window, the visit closest to 12 months was chosen.

#### HAART adherence questionnaire

Participants who had initiated HAART were asked the following questions regarding their adherence to HAART medications; “Have you taken any medications to fight HIV since your last visit?” and “Are you currently taking any medications to fight HIV?” Participants whose answer was “yes” were asked, for each medication, “How many times per day did you take this medication yesterday, 2 days ago and 3 days ago?”, and “Usually, how many times a day do you take this medication?” The possible answers to all of these questions were once, twice or three times a day or do not know. Participants who stopped HAART were also asked whether they stopped because of side effects, a belief that they no longer needed medicine or other reasons.

For this study, self reported adherence to antiretroviral therapy was dichotomized as 100% or <100% on the basis of a previously published algorithm.[14] One hundred percent adherence was defined as taking all doses as prescribed. In contrast, inclusion in the <100% adherence group resulted when the participant reported taking their current medications fewer times than prescribed.

### Statistical Methods

Baseline characteristics were analyzed using descriptive statistics. Medians and interquartile ranges were calculated for normally distributed continuous variables and proportions for categorical variables. All statistical data were analyzed using SAS Version 9.1.3 (Cary, NC).

## Results

**Figure 1:**
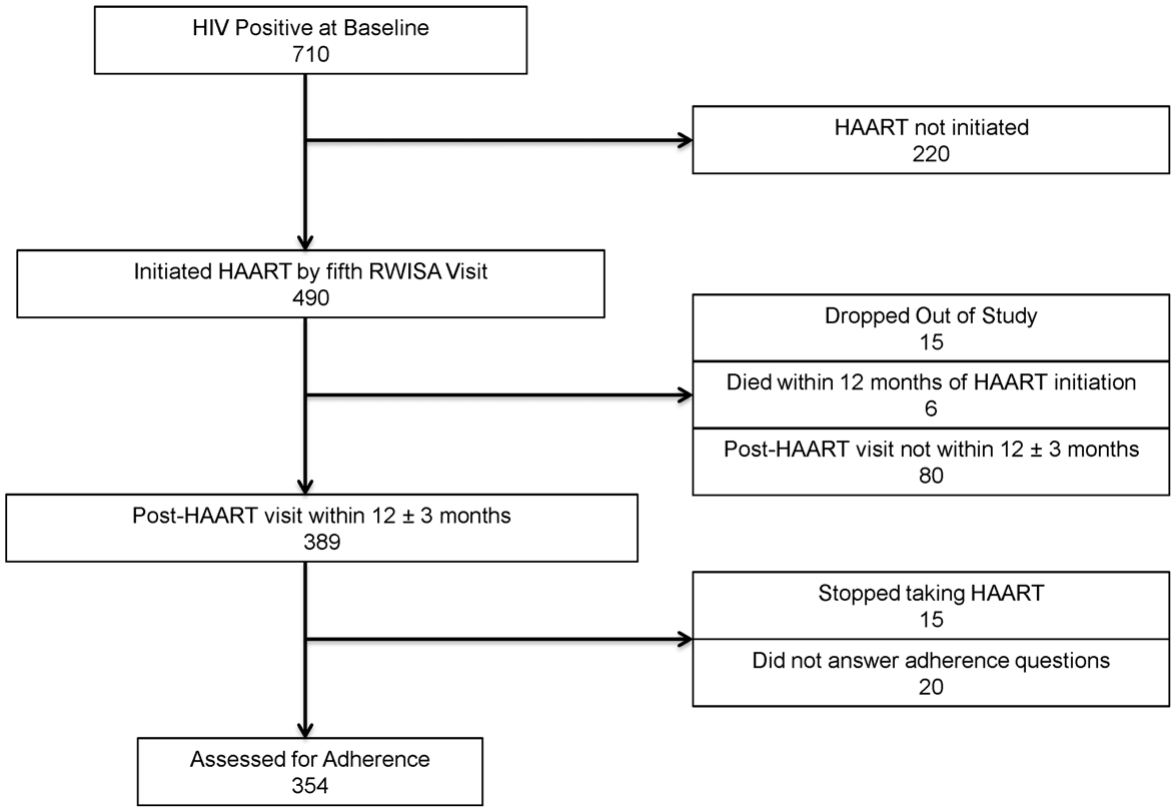
Flow diagram of RWISA Adherence Cohort. RWISA, Rwanda Women's Interassociation Study Assessment; HIV, Human immunodeficiency virus; HAART, Highly active antiretroviral therapy.

Of the 710 HIV+ women at baseline, 490 (87.2%) initiated HAART by the fifth RWISA visit. Of these 15 (3.1%) dropped out of the RWISA study; 6 (1.2%) died within 12 months after HAART initiation and 80 (19%) remained in RWISA but did not have a post-HAART initiation visit that fell within the 12±3 month time points, leaving a total of 389 women for analysis. Of these 389, 15 women were no longer taking HAART, all of whom stopped their medications without being advised to do so by their doctors. Of the remaining 374 persons who reported current HAART use, 354 responded to the adherence questions (Figure 1). Baseline characteristics of the cohort are shown in Table 1. The median age of women in the cohort was 35.5 years. Most women were poor and unemployed with little to no formal education. Many women had World Health Organization (WHO) stage 4 HIV illness defined either with or without the weight loss criterion, 56% and 42% respectively, at some point during their treatment.

**Table 1:** Participants characteristics at 12±3 months post HAART visit.

CONTINUOUS CHARACTERISTICS (n = 354 except if noted otherwise)	Median (Interquartile Range)
**Age (years)**	35.5 (32.5, 41.0)
Body Mass Index (BMI) (Kg/m^2^) (n = 349)	22.3 (20.2, 24.2)
**Days from last Pre-HAART visit to date of HAART initiation**	65.5 (20, 107)
**Days HAART initiation to closest visit to 12 months post HAART initiation**	347.5 (309, 390)
**CD4 count 0-6 months pre-HAART**	185 (128, 253)
**CD4 count 12 months Post-HAART**	264.5 (182, 380)
**MCV 0-6 months pre-HAART (n = 314)**	88 (83, 93)
**MCV 12 months post-HAART (n = 327)**	104 (98, 110)

CATEGORICAL CHARACTERISTICS (n = 354 except if noted otherwise)	n (%)
**Raped in Genocide (n = 350)**	183 (52.3%)
**Employed**	31 (8.8%)
**Had fever >2 weeks**	24 (6.8%)
**Usual number of HAART medications/day**	
1 per day	2 (0.6%)
2 per day	352 (99.4%)
**Education (n = 349)**	
No schooling	77 (22.1%)
Some primary school	128 (36.7%)
Secondary or University	144 (41.3%)
**Income per month (Frw) (1 USD = 602 Frw) (n = 351)**	
<10 K	152 (43.3%)
10–35 K	166 (47.3%)
>35 K	33 (9.4%)
**Had WHO stage 4 illness with weight loss at pre ARV**	296 (55.4%)
**Had WHO stage 4 illness without weight loss pre ARV**	149 (42.1%)
	
**Adherent to HAART medication^a^**	354 (100%)

a Based on taking the required number of doses in the previous s days according to a previously published algorithm [12]

The last visit within 6 months prior to HAART initiation tended to be a little over 2 months (median  = 65.5 days) prior to the date of initiating antiretroviral treatment. The date of the visit closest to 12 months post HAART initiation (the post-HAART visit) tended to be almost one year (median  = 347.5 days) following the start date of antiretroviral treatment. All women, 354/354, reported 100% adherence to HAART at the post-HAART visit. As adherence was 100%, further analyses of predictors of non-adherence were not undertaken.

For the 354 women who reported 100% adherence to HAART the median (interquartile range) CD4 cell count measured within 6 months prior to HAART initiation was 185 (128, 253) compared to 264.5 (182, 380) cells/mm^3^ at the post-HAART visit. Similarly, the median (interquartile range) MCV within 6 months prior to HAART initiation was 88 (83, 93) fL compared to 104 (98, 110) fL at the 12-month post-HAART initiation visit.

## Discussion

Our study indicates that self-reported adherence to antiretroviral treatment 12±3 months after initiating therapy was 100% (354/354) in this cohort of HIV-infected Rwandan women. Even considering the 15 women who had started HAART and quit taking their medications without being advised to do so by their doctors and the other 20 who did not answer the adherence questions as nonadherers, a worst-case adherence would be 91.0% (354/389). Furthermore, the attrition rate was low with only 3% (15/490) of women not returning for any follow-up visits beyond 12 months post HAART initiation. This rate of attrition is much lower than seen in other sub-Saharan African countries.[15]

Adherence in this study is higher than reported elsewhere including from other African and non-African countries.[8], [16], [17] For instance, in a study assessing factors associated with medication adherence in HIV-infected adults in Botswana, overall adherence was 81%.[16] In Nigeria, a similar study showed adherence rates to HAART of approximately 75%.[17] Our findings, however, are consistent with an earlier small study of adherence to HAART in Rwanda. A 2003 study in the Ensemble de Solidarité Therapeutique Hospitaliere En Reseau (ESTHER) antiretroviral treatment program at the Centre Hospitalier Universitaire de Kigali in Kigali, Rwanda, found that only 5% of patients reported missing a dose of their HAART medication in the 3 days prior to their adherence visit.[18] This level of adherence was confirmed using therapeutic drug monitoring in a group of 41 patients taking Triomune or Triviro (single-pill fixed dose combinations of stavudine/lamivudine/nevirapine). Only 5% of patients in this group had undetectable drug levels and 93% had levels within the therapeutic range.[18]

Our own skepticism about the high adherence in this cohort prompted us to informally compare our findings with other Rwandan health facilities. Three of the authors [SM, EM, FM] looked at HAART adherence rates by examining clinical pharmacy statistics at King Faisal Hospital Kigali (KFH, K), WE-ACTx and Gicumbi Hospital respectively. Pharmacy data from KFH, K indicated that 94% (367/400) of patients on HAART returned to refill their medications within three days of their scheduled appointment during September to December 2010, and between January to February 2011, 100% (400/400) of patients returned to fill their medications. Similarly, in the first quarter of 2010, 94% (1071/1139) of patients on HAART at the WE-ACTx clinic refilled their medications at the appropriate time. For the last quarter of 2010, 93% (2081/2237) of patients on HAART at Gicumbi Hospital appropriately refilled their medications.

Another indication that the level of adherence found in this study is accurate can be assessed by analyzing the observed changes in CD4 cell count and MCV from zero to six months prior to HAART initiation to approximately 12 months post HAART initiation. On a population level median CD4 cell counts rose about 80 cells/mm^3^ from 185 to 265 while median MCV increased about 16 fL from 88 to 104. The observed 80 cells/mm^3^ change in CD4 cell count is consistent with data from clinical trials which demonstrate that patients on HAART with good virologic control show an average increase of approximately 50–100 cells/mm^3^ per year until a steady state level is achieved.[19] The observed rise in median MCV of 16 fL from 88 to 104 may also indicate adherence to HAART for those patients taking zidovudine or stavudine. Prior studies have correlated the development of macrocytosis with zidovudine and stavudine use and suggested that simple observations of patients' MCV could be an effective method of monitoring antiretroviral adherence.[20] While we considered looking at individual changes in MCV and CD4 to see if these could identify specific non-adherers who were misreporting adherence, the repeat visit random change and measurement error of laboratory parameters such as this is so high as to negate this use of between visit changes.[21]

There are several possible reasons why adherence in this cohort of Rwandan women could be very high. Unlike many other settings, in Rwanda, when a patient becomes eligible to start antiretroviral treatment, national guidelines require that person to attend 2- to 3-day educational sessions with a “buddy” who can support his or her adherence to HAART.[22] There are mandatory social criteria for starting HAART, including acceptance to be visited by a health care worker, having a trusted person to help with compliance usually called a treatment buddy, having a fixed residence within Rwanda in a known catchment area of a health facility and disclosure of HIV status to a trusted family member. To further promote adherence, there are also follow up visits at the village level guided by a community health worker and or a member of associations of people living with HIV and AIDS.

Certain cultural aspects of Rwandan society may also contribute to high levels of HAART adherence. Following the 1994 war and genocide there has been a strong societal focus on community coherence and the sharing of meager resources. Within this communal environment the loss of a family member is a loss to the whole village or umudugudu and to a wider Rwandan community; thus, there is a strong focus on promoting health and reducing morbidity and mortality. Family members within a community are obliged by custom to support an HIV-infected person through the journey of compliance to antiretroviral therapy.

Other national characteristics specific to Rwanda may have contributed to the high adherence to HAART seen in this study. In Rwanda there is almost universal health coverage with 96% of Rwandans having health insurance, 91% of which is provided through the government-supported Mutuelle de Santé program.[22] Furthermore, in Rwanda there is a strong emphasis on personal and public health. For example, immunization coverage for children under one year of age for the six killer diseases (tuberculosis, poliomyelitis, tetanus, diphtheria, pertussis, measles) is the highest globally with 100% coverage as reported by both national statistics and UNICEF.[23] Lastly, in Rwanda, all HAART medications are provided free of charge to patients once they meet eligibility criteria for treatment initiation. The general promotion of health, almost universal insurance coverage and availability of medical care and free medications all likely contribute to better overall HAART adherence.

Some limitations of this analysis should be noted. Our study was in a cohort consisting entirely of Rwandan women and may not be generalizable to other sub-Saharan African populations. Prior studies have indicated that women may have higher HAART adherence compared to men.[17], [24], [25] This gender disparity could have been a contributing factor to the high adherence found in this study. All study participants were being followed as part of a larger research study. The majority of these participants received their HIV care at a non-governmental organization clinic where there were greater outreach efforts such as patient home visits and extra resources invested in care and treatment of women. Although there are remarkable initiatives to improve adherence even in government health care clinics, the extra resources and close follow-up for majority of patients in RWISA cohort study, could have resulted in better overall adherence compared to the general population. While only 15 of the 490 women who began HAART dropped out of the study we did not have adherence data for the 6 woman who died prior to 12±3 months post HAART initiation. There were an additional 80 women who remained in RWISA but did not have a study visit within the 12±3 months time points designated for this study. The median time from HAART initiation to follow-up for these 80 women was 639 days. Similar to the 12±3 month cohort 4 women had stopped HAART without being told to do so by their physician and the remaining 76 women reported 100% adherence. This adherence measurement occurred outside of the 12±3 months time frame set for this study so this data was not included in our analysis.

We measured HAART adherence by self-report using structured interview questions that can be subject to overestimation as patients tend to overstate their adherence to treatment. Other measures of adherence, such as pill counts, pharmacy records, electronic devices or therapeutic drug monitoring were not available to us. Even so, measuring adherence using patients' self-report can be easily replicated in most resource-limited settings including Rwanda making it a good measure for comparison. We examined the median CD4 cell count and MCV at the pre and post adherence visit to corroborate the self-reported adherence of the study participants. Given the significant variability in these between-visit hematologic parameters it is possible that individual-based changes could be due in part to underlying variability in the observed data.[21], [26], [27] However, due to the central limit theorem when comparing median CD4 cell counts (or MCV levels) in a sample size this large (354) such variability tends to cancel out, making population-based comparisons a more reliable indicator of what is happening in the population as a whole.[21] Lastly, we chose to define adherence as taking all prescribed HAART medications as directed in the three days prior to the 12±3 months adherence visit. This definition of adherence has been used in prior studies which observed substantially lower rates of adherence than we observed.[28], [29] We did not ask patients if they had missed medication doses in the month prior to their clinic visit or if they “more generally take most or nearly all of their prescribed medications”. It can be argued that this single pre-clinic visit measurement of adherence may not accurately reflect more general adherence.

In conclusion, adherence to highly active antiretroviral treatment at one year after initiation of HAART in this cohort of Rwandan women appears to be extremely high. Numerous social, cultural and political factors specific to Rwanda, and the structured required outreach to patients not coming into the pharmacy for refills, likely contribute to this high level of adherence. Future studies in Rwanda will be needed to determine the level of long-term adherence to HAART and if adherence rates change over time. More research is needed to determine which country specific factors may be contributing to the high levels of adherence to HAART in this population.
